# HBV Prevention and Treatment in Countries of Central Asia and the Caucasus

**DOI:** 10.3390/v12101112

**Published:** 2020-09-30

**Authors:** Daulet Amerzhanov, Indira Suleimenova, Salima Davlidova, Zhamilya Nugmanova, Syed Ali

**Affiliations:** 1 Nazarbayev University School of Medicine, Nur-Sultan 010000, Kazakhstan; damerzhanov@nu.edu.kz (D.A.); indira.suleimenova@nu.edu.kz (I.S.); salima.davlidova@nu.edu.kz (S.D.); 2 Kazakh National Medical University, Almaty 050000, Kazakhstan; zhamilya.nugmanova@gmail.com

**Keywords:** Central Asia, Caucasus, HBV

## Abstract

The countries of Central Asia and the Caucasus are linked by travel and trade, which is promoted by visa-free mobility across borders. Unfortunately, this migrant mobility has given rise to the transmission of various infections within this region. Overlaps in culture, tradition, and behavior among these countries provide opportunities to share experiences that have proven effective in controlling transmission. Here we present a review of hepatitis B virus (HBV) prevalence, prevention and treatment across Central Asia and the Caucasus. Overall, owing to effective measures, while HBV prevalence has been steadily declining in the region, certain gaps still exist regarding the generation and availability of HBV infection data.

## 1. Introduction

**Figure 1:**
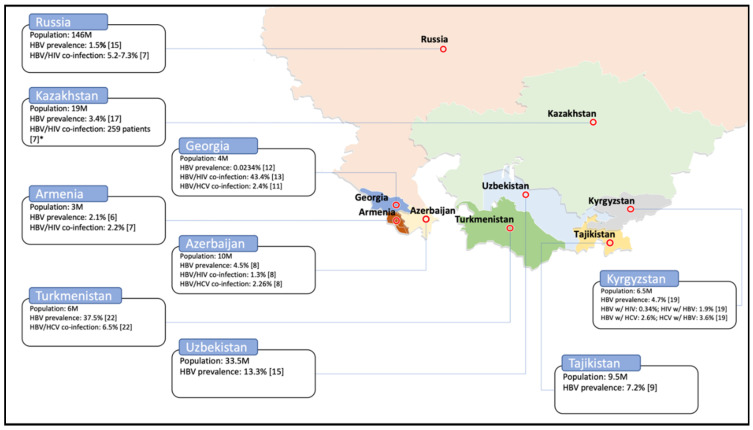
Prevalence of hepatitis B virus (HBV) and co-infections of HBV/HIV and HBV/HCV. Total population for each country [4] is mentioned along with the prevalence of single and double infections, wherever available. The map template was taken from the source [5].

Following the collapse of the Soviet Union in 1991, 15 countries gained independence: Armenia, Azerbaijan, Belarus, Estonia, Georgia, Kazakhstan, Kyrgyzstan, Latvia, Lithuania, Moldova, Russia, Tajikistan, Turkmenistan, Ukraine, and Uzbekistan [1]. Some of these countries are historically grouped based on the administrative classification used during the period of the Russian Empire. Central Asia includes Kazakhstan, Kyrgyzstan, Tajikistan, Turkmenistan, and Uzbekistan, while another sub-region is the Caucasus, which comprises Armenia, Azerbaijan and Georgia, and part of Russia also known as Northern Caucasus [2] (Figure 1). Countries in Central Asia and the Caucasus share common history leading to anthropological similarities in their societies, economic growth, and issues [3]. Open border policy within the Former Soviet Union (FSU) region promoted travel and trade on the one hand, but has, on the other, facilitated the transmission of infectious diseases. Overlaps in culture and tradition, and similarities in behavior and practices associated with infection transmission, provide opportunities to share and adapt the experiences that have proven effective in the control and treatment of infections across the region.

In this article, we provide an overview of the prevalence, control, and treatment of hepatitis B virus (HBV) infection among the nine countries of Central Asia and the Caucasus. We review the policies implemented by the governments for the prevention and treatment of HBV, and highlight areas that have worked in certain countries and may be adapted by others.

## 2. Prevalence

The prevalence of hepatitis B infection in Armenia significantly increased from <2% to 8% between 2000 and 2007 [6,7]. This may be explained by improved sensitivity of diagnostic tools or that of prevalence surveys. However, single HBV and HBV/HIV co-infection dropped to 2% in 2012 [8] and 2017 [9], respectively (Figure 1 and Table 1). In Azerbaijan, prevalence of hepatitis B infection decreased from 8% in 2007 [6] to 4.5% in 2012 [10]. Encouragingly, this number dropped to 2.8% in 2017 [11], highlighting effective implementation of routine immunization. In 2012, the HBV/HIV and HBV/HCV co-infection rates among the Azerbaijani population was estimated at 1.3% each [10]. In Georgia, the prevalence of hepatitis B was estimated at 3.4% in 2001 [12]. In a study conducted in 2005 amongst the general population, HBc antibodies and hepatitis B surface antigen (HBsAg) were found in, respectively, 11.4% and 1.1% of study participants [13]. Finally, a study in 2013 reported a significant decrease in HBV prevalence, i.e., 23.4 cases in a 100,000 population (i.e., 0.0234%) (Table 1) [14]. In Georgia, the rate of HBV/HCV co-infection was reported as 2.4% in 2005 [13]. In Georgia, a study of HBV co-infection in HIV-positive individuals showed the presence of anti-HBc and HBsAg in 43.42% and 6.86% of the population, respectively [15]. Hepatitis B prevalence in the general Kazakhstani population was reported to be 10% in 2007 [6]. In 2012, the prevalence among voluntary blood donors was estimated at 2% [16]. One year later, in 2013, this number declined to 3.8% among the general population, and to 1.8% among the Kazakhstani first-time blood donors [17]. In 2014, the prevalence among the blood donors declined to 1% [18], and in 2016, to 3.4% among the South Kazakhstan Province’s population [19]. In 2017, HBV/HIV co-infection was reported in 259 individuals. Since the sample size for this survey was not provided, it was not possible to calculate the prevalence [9]. Between 2013 and 2015, hepatitis B prevalence was estimated at 3.6% among voluntary blood donors in Kyrgyzstan [20]. This number among the general Kyrgyzstani population was assessed at 4.7% in 2015 [21], and 6.6% in 2017 [9]. In 2017, HIV prevalence among HBV patients was reported to be 0.34%, whereas HBV prevalence among HIV patients was 1.9% [20]. In patients with HBV infection, the co-infection rate with HCV was 2.6%, while in patients with HCV infection, the co-infection rate with HBV was 3.6% [20]. In the general population of the Russian Federation, hepatitis B prevalence was estimated at 1.5% in 2013 [17]. In 2016, the prevalence varied between 1% and 8% in six different Russian regions [22]. The HBV/HIV co-infection rate was assessed at between 5% and 7% in 2017 [9]. In 2017, hepatitis B prevalence in the Tajikistani general population was estimated at 7% [11], whereas among Tajik migrant workers in Russia, it was 5% in 2017 [23]. In Turkmenistan, between 2010 and 2012, hepatitis B infection was estimated to be the second most prevalent after HCV among the patients of the Center for Infectious Diseases. It was reported that 460 out of 1228 surveyed patients, i.e., 37.5%, had the HBV infection [24]. The rate of HBV/HCV co-infection among all known hepatitis patients in Turkmenistan was 6.5% between 2010 and 2012 [24]. The prevalence of hepatitis B in the general Uzbekistani population was 13% in 2013 [17], declining to >10% in 2017 [9]. The same prevalence in Uzbek migrant workers in Russia was estimated at 4% [23] (Figure 1 and Table 1). Possibly owing to improved surveillance and immunization, Azerbaijan, Georgia, Kazakhstan, Kyrgyzstan, Tajikistan, and Uzbekistan have experienced a steady decline in HBV prevalence during the last two decades (Table 1)—commendable examples for the rest of the countries in this region. These patterns are comparable to the ones reported for the European region, where HBV prevalence has been reported to vary between 0.1% to 4.4% [25].

## 3. Blood Donor Screening

As a bloodborne pathogen, HBV transmits readily through injection needles and blood transfusions. The safety of donated blood and its products is ensured by implementation of rigorous screening protocols by the blood banks [27]. According to the last reports in 2016 and 2011, blood donated in Armenia [28] and Georgia [12], respectively, was screened for hepatitis B; however, exact screening techniques used in the countries are not specified (Table 2). In Azerbaijan, donated blood products were tested for hepatitis B surface antigen (HBsAg) by 2011 [29]. According to the Kazakhstani law adopted in 2019, a laboratory study of donated blood samples for hepatitis B is carried out in two stages [30]. The first stage includes a screening for the presence of HBsAg, while the second stage involves HBV nucleic acid testing (NAT), that allows detection of the virus during its window period [30]. The second-stage testing is performed only if the first stage screening yields a negative result [30]. If the results of HBsAg serological screening and HBV nucleic acid testing are both negative, the sample is considered to be HBV-negative and acceptable for transfusion [30]. However, if the serological screening for HBsAg is positive, then two additional tests and one confirmatory test are performed. The first additional test for HBsAg is carried out using the same reagents as the previous one, while the second additional screening for HBsAg is performed using a kit from a different manufacturer [30]. The final confirmatory test is performed using an HBsAg neutralization reaction. If all three additional and confirmatory tests are negative, the blood sample is considered to be HBV-negative, however, the donor is suspended from blood donation for 6 months. Only after conducting a control test consisting of the two above-mentioned stages and receiving a negative result, is the donor allowed to donate the blood [30]. According to a WHO report in 2016, in Kyrgyzstan, HBsAg screening is performed on donated blood samples [21]. The decree adopted by the government of the Russian Federation in 2010 states that all donated blood should be screened for HBsAg [31]. If the result of the screening is positive, the serological screening is repeated two more times under the same conditions. If one of the two consecutive tests is positive, the donated blood sample should undergo confirmation with an HBsAg neutralization reaction. At the same time, each sample is screened with NAT using PCR-based tests, especially for the blood components with a short shelf life and for fresh frozen plasma that has not been quarantined. If the test is negative, it is repeated two more times under the same settings. The sample is considered positive for HBV, if at least one of the tests is positive [31]. In Tajikistan, according to government decree, all donated blood is screened for HBV using ELISA- and PCR-based testing, [27]. In Turkmenistan and Uzbekistan, no specific HBV tests are enforced by the local screening regulations adopted in 2017 [32] and 2014 [33], respectively (Table 2). Stringent regulations for blood donor screening are being implemented in Kazakhstan, Kyrgyzstan, the Russian Federation, and Tajikistan. The testing protocols employ detection of both HBsAg and HBV nucleic acid, using a multistep algorithm to ensure that all potentially HBV-infected donors are effectively screened out.

## 4. HBV Vaccination

In Armenia, the HBV vaccine was introduced in 1999, and the birth dose was instituted the same year [34]. The vaccine is monovalent, with three doses scheduled at 0, 6, and 26 weeks after birth [11]. For 2018, the vaccine coverage rate for the birth dose was estimated at 97%, while for the third dose it was estimated at 92% [34] (Table 3). In Azerbaijan, the HBV vaccine and its birth dose were implemented in 2001 [34]. The vaccine is monovalent and is given at 0, 9, and 17 weeks after birth [11]. In 2018, the hepatitis B vaccine coverage rate in Azerbaijan was 99% for the birth dose, and 95% for the third dose [34]. In Georgia, the vaccine was introduced in 2001, however the birth dose was implemented two years later, in 2003 [34]. The vaccine is scheduled at 0, 8, 12, and 16 weeks after birth [35]. A dose given at birth is monovalent, and the other three are pentavalent [35]. In 2018, the vaccine coverage rate for the birth dose was estimated at 97%, however, for the third dose it was estimated at 93% [34]. In Kazakhstan, HBV vaccination was initiated in 1998, and the birth dose was instituted in the same year [34]. The vaccine is given in three doses: a monovalent dose at birth, and tetravalent at 8 and 16 weeks after birth [36]. In 2018, the vaccine coverage rate for the birth dose was 95%, and for the third dose it was 98% in 2018 [34]. In Kyrgyzstan, the hepatitis B vaccine was implemented in 2001, while the birth dose was introduced in 1998 [34]. The vaccine is scheduled at 0, 8, 14, and 24 weeks after birth: a dose given at birth is monovalent, the other three are pentavalent [21]. In 2017, the vaccine coverage rate for the birth dose was estimated at 97%, while for the third dose at 92% [34]. In the Russian Federation, the HBV vaccine and its birth dose were implemented in 2000 [34]. The vaccine is monovalent and scheduled at 0, 4, and 24 weeks after birth [37]. However, one additional dose is recommended for children born to high-risk parents; the vaccine being scheduled at 0, 4, 8, and 48 weeks after birth [37]. High-risk parents include women who are drug abusers or HBsAg-carriers, who have hepatitis B or have suffered from it in the third trimester, those not tested for hepatitis B prenatally, or who have a family history of HBV infection [37]. In 2013, the reported vaccine coverage rate for the third dose was 97% [34]. In Tajikistan, the hepatitis B vaccine was instituted in 2002, while its birth dose was introduced in 1998 [34]. The vaccine is monovalent and is scheduled at 0, 9, and 17 weeks after birth [11]. In 2018, the vaccine coverage rate for the birth dose was estimated at 99%, and for the third dose at 96% [34] (Table 3). In Turkmenistan, the hepatitis B vaccine and its birth dose were implemented in 2002 [34]. In 2018, the vaccine coverage rate for the birth dose and the third dose was estimated at 99% [34]. The vaccine is given at 0, 8, 12, and 16 weeks after birth [38]. The dose given at birth is monovalent, while the others are pentavalent [38]. In Uzbekistan, the hepatitis B vaccine was implemented in 2001, however, its birth dose was introduced three years earlier, in 1998 [34]. The vaccine is given at 0, 8, 12, and 16 weeks after birth [39]. A dose given at birth is monovalent, while the other three doses are pentavalent [39]. In 2018, the vaccine coverage rate for the birth dose was 95%, while for the third dose it was 98% [34] (Table 3). Overall, vaccine coverage in the region appears to be satisfactory. Especially for Azerbaijan, Kazakhstan, Tajikistan, Turkmenistan, and Uzbekistan, the coverage of birth and the third dose is reported, impressively, to be between 95% and 99%.

## 5. Treatment for HBV

With the exception of Georgia and Turkmenistan, for all the countries of Central Asia and the Caucasus, clinical guidelines for the treatment of HBV infection are either available online or in the report of the Alliance for Public Health [9] (Table 4). Lists of the antiviral medications used in each country are available online, except for Turkmenistan. According to a 2015 WHO report, the drugs approved for the treatment of the chronic hepatitis B infection include: lamivudine, adefovir, entecavir, telbivudine, tenofovir, emtricitabine, and standard and pegylated interferon (PEG-IFN) [40]. However, according to WHO, standard and pegylated interferon are not recommended for the low- and middle-income countries, due to their high cost. Interestingly, contrary to this recommendation, all the Central Asian and the Caucasus countries, except for Azerbaijan and Kyrgyzstan (where an official clinical protocol is not available), use interferon as the antiviral medication (Table 4). Aside from Turkmenistan, all the countries use at least one of the nucleos (t) ide inhibitors recommended by WHO.

## 6. Conclusions

Overall, in Central Asia and the Caucasus, the prevalence of HBV and associated co-infections has been declining, owing to rigorous implementation of vaccination, treatment, and blood donor screening protocols. Thorough and large-scale surveys are key to recording evolving trends in the prevalence and transmission of infections. While regular surveys are being carried out by countries such as Armenia, Azerbaijan, Georgia, and Kazakhstan, certain exceptions still exist. Another area of focus is to enhance public accessibility of the information related to data, as well as policies regarding HBV infection and its control. For the prevalence of HBV single and co-infection, information was readily available from Azerbaijan, Georgia, and Kyrgyzstan, while from the rest of the countries this information was either partially available or unavailable. Additionally commendable is the full disclosure of information regarding blood-screening protocols by Kazakhstan, Kyrgyzstan, the Russian Federation, and Tajikistan. It appears, therefore, that in certain countries the information, while it may exist, is not readily available for review or scrutiny, compromising the transparency of the process.

## Figures and Tables

**Table 1:** Prevalence of HBV and co-infections of HBV/HIV and HBV/HCV. Prevalence of single and double infections, wherever available, is mentioned along with the source. In all the studies, prevalence was measured by the presence of hepatitis B surface antigen (HBsAg) in the blood. * Prevalence of anti-HBc antibodies in HIV-positive individuals. ** Prevalence of HBsAg in HIV-positive individuals. *** The source mentions the number of HBV/HIV infected patients without mentioning the sample size. n/a, not available.

Country	HBV		Co-Infection HBV/HIV		Co-Infection HBV/HCV	
	Prevalence	Year	Prevalence	Year	Prevalence	Year
Armenia	2.1%	2012 [8]	2.2%	2017 [9]	n/a	n/a
	8%	2007 [6]				
	<2%	2000 [7]				
Azerbaijan	2.8%	2017 [11]	1.3%	2012 [10]	2.26%	2012 [10]
	4.5%	2012 [10]				
	0.02%	2010 [26]				
	8%	2007 [6]				
Georgia	0.0234%	2013 [14]	43.42% *	2008 [15]	2.4%	2005 [13]
	1.1%	2005 [13]				
			6.86% **	2008 [15]		
	3.4%	2001 [12]				
Kazakhstan	3.4%	2016 [19]	*n* = 259 ***	2017 [9]	n/a	n/a
	1.12%	2015 [18]				
	2.1%	2015 [16]				
	1.8%	2014 [17]				
	3.8%					
	10%	2007 [6]				
Kyrgyzstan	4.7%	2017 [21]	HBV w/HIV: 0.34% HIV w/HBV: 1.9%	2017 [20]	HBV w/HCV: 2.6% HCV w/HBV: 3.6%	2017 [20]
	3.6%	2017 [20]				
	6.6%	2017 [9]				
Russian Federation	1.2–8.2%	2016 [22]	5.2–7.3%	2017 [9]	n/a	n/a
	1.5%	2014 [17]				
Tajikistan	5.3%	2017 [23]	n/a	n/a	n/a	n/a
	7.2%	2017 [11]				
Turkmenistan	37.5%	2018 [24]	n/a	n/a	6.5%	2018 [24]
Uzbekistan	4.1%	2017 [23]	n/a	n/a	n/a	n/a
	>10%	2017 [9]				
	13.3%	2014 [17]				

**Table 2:** Screening for HBV in blood donors. Sources of information are mentioned in parenthesis; n/a, not available.

Country	Is Screening Performed?	Screening
Armenia	Yes [28]	Not specified.
Azerbaijan	Yes [29]	HBsAg screening.
Georgia	Yes [12]	Not specified.
Kazakhstan	Yes [30]	Screening for HBsAg is performed: If HBsAg is present, blood is tested again for HBsAg under the same conditions, and with test kit from a different manufacturer (different conditions) Confirmatory test for HBsAg neutralization If HBsAg is negative, HBV NAT is performed
Kyrgyzstan	Yes [21]	HBsAg screening.
Russian Federation	Yes [31]	Screening for HBsAg is performed: If HBsAg is present, the test is repeated two times under the same conditions If at least one test is positive, the sample is confirmed with HBsAg neutralization reaction Simultaneously, each sample is screened with PCR-based tests. This test is performed for blood components with short shelf life and fresh frozen plasma that was not quarantined: If negative, the test is performed 2 times with the same settings. If at least one test is positive, sample is considered HBV positive
Tajikistan	Yes [27]	Not specified. National program is aimed to introduce both ELISA- and PCR-based test systems.
Turkmenistan	Yes [32]	n/a

**Table 3:** Implementation of HBV vaccination in the countries of Central Asia and the Caucasus. All the data were retrieved from the source [34], except for the information on vaccine type and schedule, for which the sources of information are mentioned in parenthesis.

Country	Year of Vaccine Introduced in Entire Country	Year of Birth Dose Introduced	Coverage of Birth Dose in 2018, %	Coverage of 3rd Dose in 2018, %	Type of Vaccine/Schedule in Weeks
Armenia	1999	1999	97	92	Monovalent vaccine given at 0, 6, 26 weeks [11].
Azerbaijan	2001	2001	99	95	Monovalent vaccine given at 0, 9, 17 weeks [11].
Georgia	2001	2003	97	93	Monovalent vaccine given at birth [35]; Pentavalent vaccine given at 8, 12, 16 weeks [35].
Kazakhstan	1998	1998	95	98	Monovalent vaccine given at birth [36]; Tetravalent vaccine given at 8 and 16 weeks [36].
Kyrgyzstan	2001	1998	97	92	Monovalent vaccine given at birth [21]; Pentavalent vaccine given at 8, 14, 24 weeks [21].
Russian Federation	2000	2000	n/a	97	Monovalent vaccine given at 0, 4, 24 weeks OR at 0, 4, 8, 48 weeks [37].
Tajikistan	2002	1998	99	96	Monovalent vaccine given at 0, 9, 17 weeks [11].
Turkmenistan	2002	2002	99	99	Monovalent vaccine given at birth [38]; Pentavalent vaccine given at 8, 12, 16 weeks [38].
Uzbekistan	2001	1998	95	98	Monovalent vaccine given at birth [39]; Pentavalent vaccine given at 8, 12, 16 weeks [39].

**Table 4:** Implementation of HBV treatment in the countries of Central Asia and the Caucasus. n/a, not available.

Country	Implementation of Treatment Protocol	Antiviral Medications Available in the Country
Armenia	Yes [9]	Interferon alpha, pegylated interferon, lamivudine [41]
Azerbaijan	Yes [9]	Lamivudine, lamivudine generic, tenofovir [9]
Georgia	n/a	Interferon alpha, pegylated interferon, lamivudine, adefovir dipivoxil and tenofovir [41]
Kazakhstan	Yes [9]	Pegylated interferon alpha, tenofovir disoproxil fumarate, tenofovir alafenamide fumarate, entecavir [42]
Kyrgyzstan	Yes [9]	Lamivudine generic, entecavir generic, tenofovir, tenofovir generic, emtricitabine, emtricitabine generic [9]
Russian Federation	Yes [9]	Pegylated interferon alpha, lamivudine, entecavir, tenofovir, telbivudine [43]
Tajikistan	Yes [9]	Interferon alpha, pegylated interferon, adefovir, entecavir, emtricitabine, lamivudine, tenofovir, telbivudine [44]
Turkmenistan	n/a	n/a
Uzbekistan	Yes [9]	- Interferon alpha, pegylated interferon [44] - Lamivudine generic, tenofovir generic, emtricitabine generic [9]
